# Usefulness of the endotoxin activity assay as a biomarker to assess severity in ICU patients

**DOI:** 10.1186/cc13402

**Published:** 2014-03-17

**Authors:** M Noguchi, T Ikeda, K Ikeda, S Suda, A Kodaira, T Ueno, A Ohmi

**Affiliations:** 1 Tokyo Medical University, Tokyo, Japan

## Introduction

Endotoxin is a component of the membrane of Gram- negative bacteria and plays a key role in the pathogenesis of sepsis[1]. Measurement of endotoxin levels in patient blood is important for the early diagnosis of and appropriate determination of the treatment strategy for sepsis. The aim of this study was to investigate the prevalence of endotoxemia in Japanese criticallyill patients using the endotoxin activity assay (EAA), a newly developed rapid assay of endotoxin. Blood endotoxin levels (EA levels) of 314 patients admitted to our university hospital ICU were measured within 24 hours from admission, and their correlation with disease severity and outcome was examined.

## Methods

The study is a single-center retrospective analysis of critically ill patients admitted to our university hospital ICU from November 2006 to March 2012. All patients whose eA and procalcitonin levels were measured and severity criteria of disease recorded were enrolled. A total of 314 patients were analyzed.

## Results

The mean ± SD of all ICU-admitted patients (*n *= 314) was 0.39 ± 0.25, and that of healthy volunteers (*n *= 61) was 0.10 ± 0.09. The mean APACHE II score at admission increased in parallel with increased EA levels. The mean (± SD) APACHE II score in the very low group of patients (EA <0.2) was 17.3 ± 8.9, while that in the low group (0.2 ≤EA <0.4) was 20.6 ± 9.2; it was 22.6 ± 8.1 in the intermediate group (0.4 ≤EA <0.6) and 25.3 ± 8.5 in the high group (0.6 ≤EA). The difference between the groups was statistically significant. The difference between the groups was statistically significant. The percentages of patients diagnosed with severe sepsis or septic shock were 19.3%, 34.5%, 50.0% and 81.3% in the very low, low, intermediate and high groups, respectively.

## Conclusion

Our patients' EA levels were significantly correlated with disease severity criteria and 28-day mortality of the patients. When the EA level and procalcitonin level were used concomitantly, disease severity could be assessed more precisely than when either marker was used alone. These results suggest that the EA level is a useful marker for disease severity assessment and outcome prediction in critically ill patients.
